# Supplementary material for: Sleep-Body Composition Relationship: Roles of Sleep Behaviors in General and Abdominal Obesity in Chinese Adolescents Aged 17–22 Years
Source: Nutrients. 2023 Sep 25;15(19):4130. doi: 10.3390/nu15194130 (PMC10574676; doi:10.3390/nu15194130)
Supplement: Supplementary file 1 [file nutrients-15-04130-s001.zip › nutrients-2547197-supplementary.pdf]

# Sleep-body Composition Relationship: Roles of Sleep Behaviors in General and Abdominal Obesity in Chinese Adolescents Aged 17-22 years

**Yalin Song, Lu Gong, Xiaomin Lou, Huijun Zhou, Yudan Hao, Qiuyuan Chen, Yize Zhao, Xili Jiang, Lijie Li and Xian Wang ***

**Table S1.** Description of body composition parameters according to demographic characteristics of study population.

| Variables | Gender | | Race | | Location | | The only child | | Father's education | | Mother's education | | Self-rated family income | |
|---|---|---|---|---|---|---|---|---|---|---|---|---|---|---|
| | *t* | *P* | *t* | *P* | *t* | *P* | *t* | *P* | *F* | *P* | *F* | *P* | *F* | *P* |
| Visceral fat area (cm$^2$) | −5.468 | **<0.001** | 0.803 | 0.422 | −0.768 | 0.443 | −1.648 | 0.100 | 4.765 | **0.001** | 3.189 | **0.013** | 1.460 | 0.233 |
| Body mass index (kg/m$^2$) | 1.536 | 0.125 | 0.483 | 0.629 | 0.023 | 0.982 | −0.784 | 0.433 | 2.700 | **0.030** | 2.585 | **0.037** | 0.168 | 0.846 |
| Waist to height ratio | −3.221 | **0.001** | 0.616 | 0.538 | 0.216 | 0.829 | −0.471 | 0.638 | 3.287 | **0.011** | 2.308 | 0.057 | 0.310 | 0.734 |
| Waist to hip ratio | 0.337 | 0.737 | 0.653 | 0.514 | −0.182 | 0.856 | −1.052 | 0.293 | 4.920 | **0.001** | 2.344 | 0.054 | 1.599 | 0.203 |
| Fat mass index | −9.637 | **<0.001** | 0.667 | 0.505 | 0.104 | 0.917 | −0.517 | 0.605 | 3.182 | **0.014** | 2.117 | 0.078 | 0.532 | 0.588 |
| Fat free mass index | 18.284 | **<0.001** | −0.103 | 0.918 | −0.432 | 0.666 | −0.804 | 0.422 | 0.559 | 0.693 | 1.375 | 0.242 | 1.131 | 0.324 |
| Body fat percentage (%) | −18.554 | **<0.001** | 0.767 | 0.443 | 0.082 | 0.934 | −0.556 | 0.578 | 2.630 | **0.034** | 1.630 | 0.166 | 1.168 | 0.312 |

Tests for difference were performed by using unpaired *t*-test and univariate one-way ANOVAs. Bold: $p$<0.05.

**Table S2.** Description of body composition parameters according to takeaway food consumption, breakfast consumption, vegetables consumption, fruits consumption and dried fruits consumption of study population.

| Variables | Takeaway food consumption/week | | Breakfast consumption/week | | Vegetables consumption/d | | Fruits consumption/d | | Dry fruits consumption/d | |
|---|---|---|---|---|---|---|---|---|---|---|
| | *F* | *P* | *t* | *P* | *t* | *P* | *F* | *P* | *F* | *P* |
| Visceral fat area (cm$^2$) | 0.548 | 0.584 | −2.397 | **0.017** | 0.084 | 0.933 | −0.178 | 0.859 | 0.832 | 0.406 |
| Body mass index (kg/m$^2$) | 1.258 | 0.209 | −1.218 | 0.224 | −0.187 | 0.852 | 1.572 | 0.117 | 0.846 | 0.398 |
| Waist to height ratio | 0.576 | 0.565 | −1.251 | 0.211 | 0.445 | 0.657 | 0.765 | 0.445 | 0.668 | 0.505 |
| Waist to hip ratio | −1.137 | 0.256 | −0.203 | 0.840 | 0.591 | 0.555 | 0.559 | 0.576 | 0.508 | 0.612 |
| Fat mass index | 1.228 | 0.220 | −2.324 | **0.021** | 0.353 | 0.724 | −0.426 | 0.670 | 0.910 | 0.363 |
| Fat free mass index | 0.453 | 0.651 | 0.816 | 0.415 | −0.726 | 0.468 | 3.357 | **0. 001** | −0.087 | 0.931 |
| Body fat percentage (%) | 1.195 | 0.233 | −2.589 | **0.010** | 0.808 | 0.420 | −1.466 | 0.143 | 0.946 | 0.345 |

Tests for difference were performed by using unpaired *t*-test and univariate one-way ANOVAs. Bold: $p$<0.05.

**Table S3.** Description of body composition parameters according to pure juice consumption, soft drinks consumption and sugar-sweetened beverage consumption of study population.

| Variables | Pure juice consumption (>250ml)/d | | Soft drinks consumption (⩾250ml)/week | | Sugar-sweetened beverage Consumption (⩾250ml)/week | |
|---|---|---|---|---|---|---|
| | *F* | *P* | *F* | *P* | *F* | *P* |
| Visceral fat area (cm²) | 0.535 | 0.593 | 1.322 | 0.187 | 0.657 | 0.511 |
| Body mass index (kg/m²) | −0.315 | 0.753 | −0.013 | 0.989 | 1.239 | 0.216 |
| Waist to height ratio | 0.901 | 0.368 | 0.375 | 0.708 | 1.096 | 0.273 |
| Waist to hip ratio | 0.550 | 0.582 | −0.693 | 0.488 | 0.052 | 0.959 |
| Fat mass index | 1.381 | 0.168 | 2.038 | **0.042** | 0.989 | 0.323 |
| Fat free mass index | −2.356 | **0.019** | −3.091 | **0.002** | 0.752 | 0.452 |
| Body fat percentage (%) | 2.172 | **0.030** | 2.927 | **0.004** | 1.022 | 0.307 |

Tests for difference were performed by using univariate one-way ANOVAs. Bold: $p<0.05$.

**Table S4.** Description of body composition parameters according to weekday screen time and weekend screen time of study population.

| Variables | Weekday screen time/d | | Weekend screen time/d | |
|---|---|---|---|---|
| | *F* | *P* | *F* | *P* |
| Visceral fat area (cm²) | 0.268 | 0.789 | −0.476 | 0.634 |
| Body mass index (kg/m²) | 0.615 | 0.539 | −0.854 | 0.394 |
| Waist to height ratio | 0.400 | 0.689 | −0.976 | 0.330 |
| Waist to hip ratio | −0.363 | 0.717 | −1.144 | 0.253 |
| Fat mass index | 0.312 | 0.755 | −0.592 | 0.554 |
| Fat free mass index | 0.918 | 0.360 | −0.573 | 0.567 |
| Body fat percentage (%) | 0.380 | 0.704 | | 0.936 |

Tests for difference were performed by using univariate one-way ANOVAs.

**Table S5.** Description of body composition parameters according to duration of physical exercise each time and number of physical exercise of study population.

| Variables | Duration of physical exercise each time | | Number of physical exercise/d | |
|---|---|---|---|---|
| | *t* | *P* | *t* | *P* |
| Visceral fat area (cm²) | −0.671 | 0.507 | −1.393 | 0.164 |
| Body mass index (kg/m²) | −2.014 | **0.045** | −0.818 | 0.414 |
| Waist to height ratio | −0.959 | 0.338 | −0.986 | 0.325 |
| Waist to hip ratio | −1.600 | 0.119 | −0.201 | 0.840 |
| Fat mass index | 0.467 | 0.640 | −1.574 | 0.116 |
| Fat free mass index | −4.324 | **<0.001** | 0.525 | 0.600 |
| Body fat percentage (%) | 1.903 | 0.058 | −1.910 | 0.057 |

Tests for difference were performed by using unpaired *t*-test. Bold: $p<0.05$.

**Table S6.** Description of body composition parameters according to alcohol consumption and smoking of study population.

| **Variables** | **Alcohol consumption** | | **Smoking** | |
|---|---|---|---|---|
| | ***t*** | ***P*** | ***t*** | ***P*** |
| Visceral fat area (cm$^2$) | 1.196 | 0.233 | 2.060 | **0.040** |
| Body mass index (kg/m$^2$) | −0.628 | 0.530 | 0.093 | 0.926 |
| Waist to height ratio | 0.515 | 0.607 | 1.468 | 0.143 |
| Waist to hip ratio | −0.777 | 0.438 | 1.617 | 0.107 |
| Fat mass index | 2.073 | **0.039** | 2.223 | **0.027** |
| Fat free mass index | −4.037 | **<0.001** | −2.844 | **0.005** |
| Body fat percentage (%) | 3.372 | **0.001** | 3.125 | **0.002** |

Tests for difference were performed by using unpaired *t*-test. Bold: $p<0.05$.
